# Risk factors, impact and treatment of postoperative lymphatic leakage in children with abdominal neuroblastoma operated on by laparotomy

**DOI:** 10.1186/s12893-024-02459-3

**Published:** 2024-05-29

**Authors:** Jun Feng, Jianing Mou, Shen Yang, Qinghua Ren, Saishuo Chang, Wei Yang, Haiyan Cheng, Xiaofeng Chang, Zhiyun Zhu, Jianyu Han, Hong Qin, Huanmin Wang, Xin Ni

**Affiliations:** 1 Department of Surgical Oncology, Beijing Children’s Hospital, Capital Medical University, National Center for Children’s Health, Beijing, 100045 China; 2 Children’s Hospital, Capital Institute of Pediatrics, 2# Yabao Road, Chaoyang District, Beijing, 100020 China; 3 Beijing Children’s Hospital, National Center for Pediatric Cancer Surveillance, Capital Medical University, National Center for Children’s Health, Beijing, 100045 China

**Keywords:** Children, Neuroblastoma, Lymphatic leakage, Treatment strategy, Risk factors

## Abstract

**Background:**

Lymphatic leakage is one of the postoperative complications of neuroblastoma. The purpose of this study is to summarize the clinical characteristics and risk factors of lymphatic leakage and try to find effective prevention and treatment measures.

**Methods:**

A retrospective study included 186 children with abdominal neuroblastoma, including 32 children of lymphatic leakage and 154 children of non-lymphatic leakage. The clinical information, surgical data, postoperative abdominal drainage, treatment of lymphatic leakage and prognosis of the two groups were collected and analyzed.

**Results:**

The incidence of lymphatic leakage in this cohort was 14% (32 children). Through univariate analysis of lymphatic leakage group and non-lymphatic leakage group, we found that lymphatic leakage increased the complications, prolonged the time of abdominal drainage and hospitalization, and delayed postoperative chemotherapy (*p* < 0.05). In this cohort, the median follow-up time was 46 (95% CI: 44–48) months. The follow-up data of 7 children were partially missing. 147 children survived, of which 23 had tumor recurrence (5 children recurred in the surgical area). 37 children died, of which 32 had tumor recurrence (9 children recurred in the operation area). In univariate analysis, there was no statistical difference in overall survival (*p* = 0.21) and event-free survival (*p* = 0.057) between lymphatic leakage group and non-lymphatic leakage group, while 3-year cumulative incidence of local progression was higher in lymphatic leakage group (*p* = 0.015). However, through multivariate analysis, we found that lymphatic leakage did not affect event-free survival, overall survival and cumulative incidence of local progression in children with neuroblastoma. Resection of 5 or more lymphatic regions was an independent risk factor for lymphatic leakage after neuroblastoma surgery. All 32 children with lymphatic leakage were cured by conservative treatment without surgery. Of these, 75% (24/32) children were cured by fat-free diet or observation, 25% (8/32) children were cured by total parenteral nutrition. The median drain output at diagnosis in total parenteral nutrition group was higher than that in non-total parenteral nutrition group (*p* < 0.001). The cut-off value was 17.2 ml/kg/day.

**Conclusions:**

Lymphatic leakage does not affect the prognosis of children with neuroblastoma, but long-term drain output caused by lymphatic leakage will still adversely affect postoperative complications and follow-up treatment, which requires attention and active treatment measures. More attention should be paid to the children with 5 or more lymphatic regions resection, and the injured lymphatic vessels should be actively found and ligated after tumor resection to reduce the postoperative lymphatic leakage. Early application of total parenteral nutrition is recommended for those who have drain output at diagnosis of greater than 17.2 ml/kg/day.

**Level of evidence:**

Level III, Treatment study (Retrospective comparative study).

**Supplementary Information:**

The online version contains supplementary material available at 10.1186/s12893-024-02459-3.

## Abstract (highlights)

**What is already known on this topic** –*Lymphatic leakage is one of the postoperative complications of neuroblastoma, and may affect the follow-up treatment and prognosis of children.*

**What this study adds** –*Lymphatic leakage affects the follow-up treatment of children with NB, but does not affect the prognosis. Lymphatic leakage may occur when 5 or more lymphatic regions are removed. When drain output exceeds 17.2 ml/kg/day at diagnosis, TPN should be used.*

**How this study might affect research, practice or policy** –*Surgeons can predict the occurrence of lymphatic leakage after NB surgery and apply more effective treatment.*

**Supplementary Information:**

The online version contains supplementary material available at 10.1186/s12893-024-02459-3.

## Introduction

Neuroblastoma (NB) is the most common extracranial malignant solid tumor in children [1, 2]. The occurrence of abdominal NB is unknown, and most of the tumors are large, accompanied by lymph node metastasis around the tumor or distant metastasis. At present, chemotherapy, surgery, radiotherapy, autologous stem cell transplantation and immunotherapy are commonly used to treat NB. However, local tumors often fuse with the surrounding metastatic lymph nodes to wrap around retroperitoneal vessels, resulting in difficult surgery and many complications. Nevertheless, studies have shown that complete tumor resection and adequate lymph node resection can improve the prognosis of medium-and high-risk children [3–6]. Therefore, surgery is still an important method in the treatment of NB.

In recent years, with the widespread implementation of neuroblastoma-related radical lymph node dissection, lymphatic leakage also may occur after surgery. It directly leads to an increase in abdominal drainage and prolongs the hospitalization time of some patients. Moreover, long-term lymphatic leakage and abdominal drainage may also cause some complications, affect postoperative chemotherapy, and even ultimately affect the prognosis [7, 8]. Therefore, how to prevent and treat lymphatic leakage has become a problem to be solved in NB.

At present, there are few studies on lymphatic leakage after abdominal NB surgery in children [7–12]. In this study, we conducted a retrospective study on children with abdominal NB, summarized the clinical characteristics and treatment experience of lymphatic leakage, and analyzed the impact of lymphatic leakage on prognosis and the risk factors of its occurrence, trying to find effective prevention and treatment measures.

## Materials and methods

### Patients

This is a retrospective case-control study from a single center. A total of 298 children with NB who were treated in the Department of Oncology of Beijing Children’s Hospital from April 2018 to March 2020 in the NB database of our center were selected as subjects. The inclusion criteria were children with abdominal NB who had completed their first surgical treatment in our center. The exclusion criteria included children who were not operated on or operated on at other hospitals, children with NB in neck, mediastinum and pelvis, children with secondary surgery, children with pancreatic fistula, pancreatic injury, biliary fistula, intestinal fistula, urine leakage, chylothorax and major ascites of unknown cause after surgery (Fig. S1). Our study has been approved by the Ethics Committee of Beijing Children’s Hospital ([2022]-E-212-R), and informed consent was obtained from the guardians of all children.

**Fig. 1:**
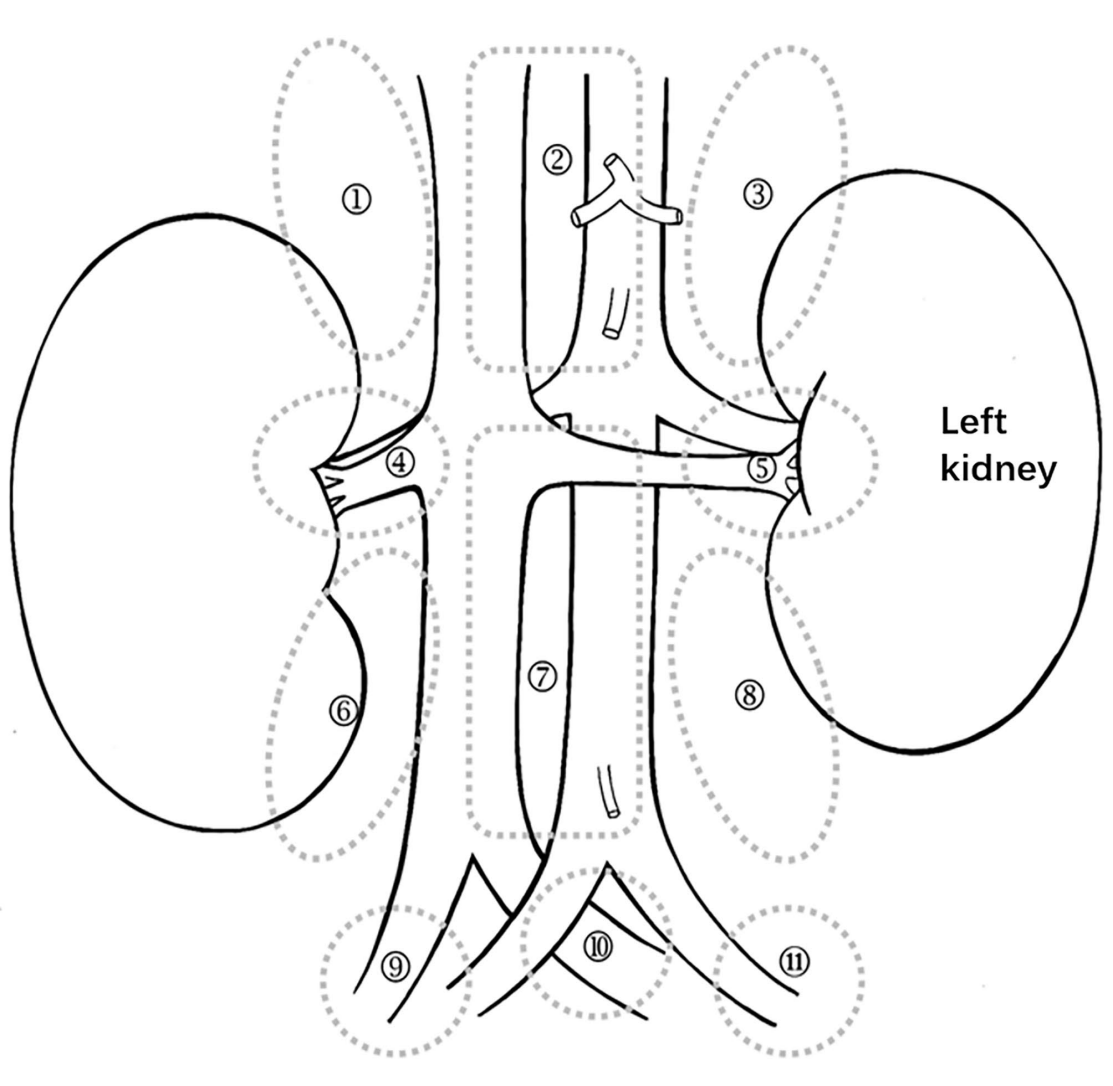
Schematic diagram of the retroperitoneal lymphatic region: ①Right-upper region, Includes the lymph nodes on the right side of the inferior vena cava and above the right renal artery; ②Middle-upper region, Includes the lymph nodes above the level of the renal artery and between the inferior vena cava and the abdominal aorta, and the lymph nodes around the celiac trunk and the superior mesenteric artery; ③Left-upper region, Includes the lymph nodes on the left side of the abdominal aorta and above the left renal artery; ④Right renal vessel, Includes lymph nodes around the arteries and veins of the right kidney; ⑤Left renal vessel, Includes lymph nodes around the arteries and veins of the left kidney; ⑥Right-lower region, Includes the lymph nodes on the right side of the inferior vena cava and below the right renal artery; ⑦Middle-lower region, Includes the lymph nodes below the level of renal artery, above the level of iliac vessels and between the inferior vena cava and abdominal aorta, and the lymph nodes around the inferior mesenteric artery; ⑧Left-lower region, Includes the lymph nodes on the left side of the abdominal aorta and below the left renal artery; ⑨Right iliac vessel, Includes the lymph nodes around the right iliac artery and vein; ⑩Bifurcation of the abdominal aorta, Includes the lymph nodes between the bilateral iliac vessels; ⑪Left iliac vessel, Includes the lymph nodes around the left iliac artery and vein

Tumor stage and risk group were evaluated according to the International Neuroblastoma Risk Group (INRG). Eleven lymphatic regions were identified according to the anatomical distribution of the retroperitoneal lymphoid system (Fig. 1) [13]. The preoperative imaging results were used to evaluate whether the tumor crossed the midline and whether there were image-defined risk factors (IDRFs) [14, 15]. The number of important retroperitoneal vessels invaded by the tumor was obtained according to the surgical records, and the degree of tumor resection and the number of resected lymphatic regions were evaluated. Overall survival (OS) calculated the time from diagnosis to death or last follow-up. Event-free survival (EFS) calculated the time from diagnosis to recurrence at any site or death from any cause or last follow-up. Cumulative incidence of local progression (CILP) calculated the time from diagnosis to local tumor recurrence or progression, regardless of metastatic lesions.

### The process of NB operation and treatment of lymphatic leakage

**Fig. 2:**
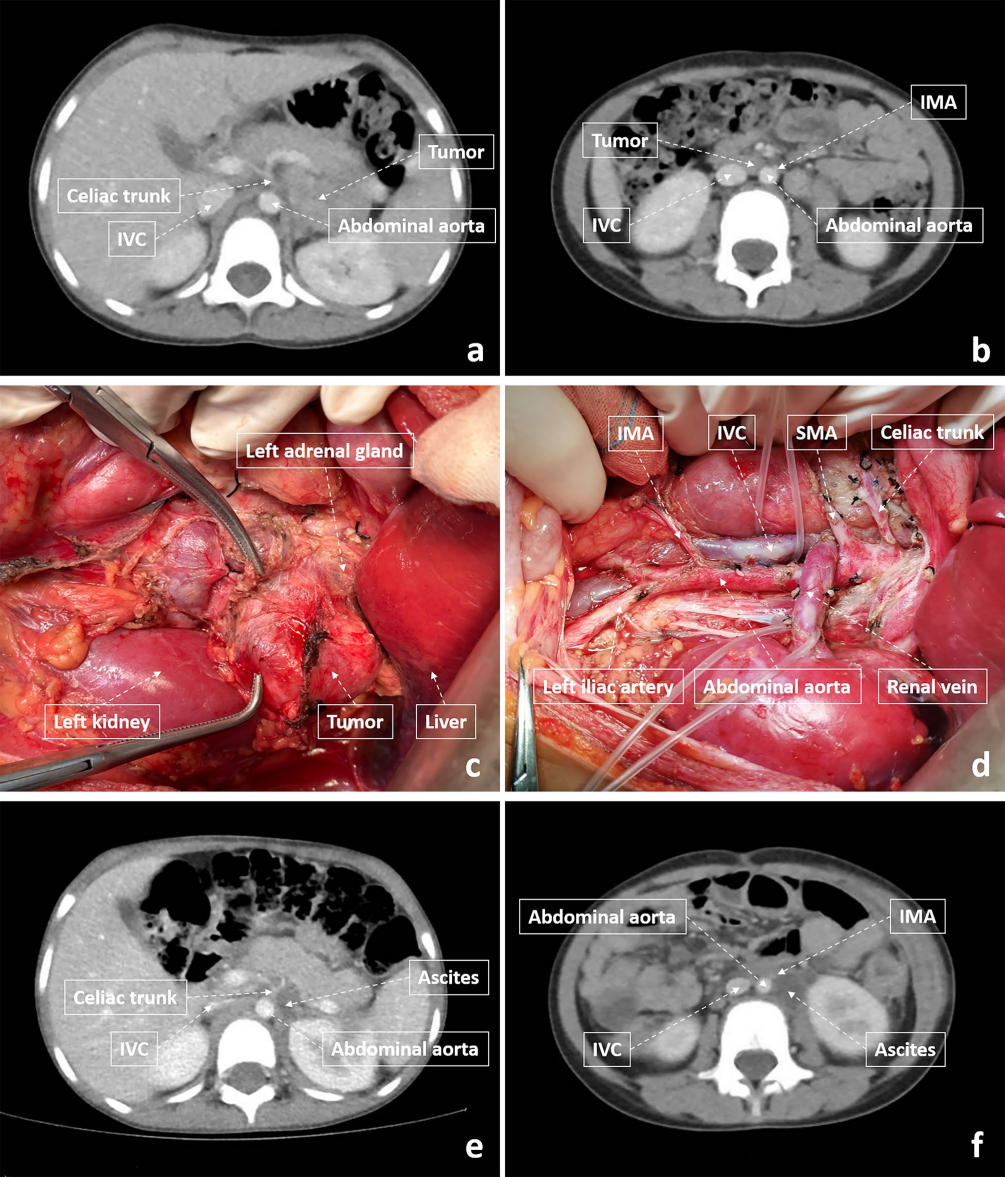
The preoperative, operative and postoperative images of NB. (**a-b**) Preoperative CT scan showed that the tumor wrapped around retroperitoneal vessels. (**c-d**) Operative photos before, and after adrenalectomy with lymphatic dissection. (**e-f**) The CT scan of one month after operation.

The surgical treatment of all the children in this study was completed by a team at the Department of Oncology of Beijing Children’s Hospital. Resection of the primary tumor and metastatic lymph nodes was performed according to imaging and intraoperative findings. When the tumor wraps around or invades the retroperitoneal vessels, vascular skeletalization was used to remove the tumor (Fig. 2) [16–18]. After the tumor was removed, the injured lymphatic vessels of mesentery and retroperitoneum were ligated or repaired by silk suture. After confirming that there was no obvious exudation in the operative area, bioprotein glue was routinely sprayed in the operative area. In open surgery, we routinely place a retroperitoneal drainage tube at the end of the operation. According to our experience, laparoscopic surgery could be considered if the localized tumor was confined to one body compartment and did not involve the list of IDRFs [19]. And if the localized tumor did not invade the surrounding tissue and no more than 2 lymphatic regions were resected, the drainage tube was generally not considered in laparoscopic surgery.

The amount and characteristics of abdominal drainage were recorded every day after the operation. Lymphatic leakage is defined as chylous drainage (milky-white appearance of the fluid) [7–9]. According to the definition, the children were divided into the lymphatic leakage group and the non-lymphatic leakage group. Most children with lymphatic leakage were treated with fat-free diet first after diagnosed. Children with malnutrition were treated with fat-free diet and medium chain triglycerides (MCT). When fat-free diet with or without MCT was effective (drainage was reduced to about 50 ml/day), abdominal drainage was removed. When fat-free diet with or without MCT was ineffective (drainage increases or did not decrease), it was changed to total parenteral nutrition (TPN). TPN could be discontinued and fat-free diet could be restored when drainage was reduced to about 100 ml/day. When the drainage reduced to about 50 ml/day, abdominal drainage was removed. If there wasn’t a large amount of ascites or progressive encapsulated effusion by ultrasound and/or computerized tomography, lymphatic leakage was considered to be cured. Only a few children of lymphatic leakage with little or much drainage were first received the treatment of observation or TPN.

Postoperative chemotherapy is usually carried out within 3 weeks after the operation according to the INRG. The follow-up treatment measures, and prognosis of the children were obtained through outpatient reexamination or telephone consultation.

### Data analysis and statistics

The clinical information, surgical data, pathological results, postoperative abdominal drainage, postoperative complications, hospital stay, drainage duration, treatment and prognosis of the lymphatic leakage and non-lymphatic leakage groups were collected and analyzed retrospectively.

Statistical analysis was performed by using SPSS 26.0. Continuous variables were presented as the mean with standard deviation or median and interquartile range if the normality hypothesis test rejected the null hypothesis of the normal distribution. Categorical variables were reported as counts and percentages. Two independent samples t tests and χ2 tests were used to compare characteristics between the lymphatic leakage and non-lymphatic leakage groups. The daily amount of chylous drainage per kilogram of children with lymphatic leakage was calculated, and the difference of the amount of chylous drainage between non-TPN and TPN groups was compared by two independent sample t tests. Receiver operating characteristic (ROC) curve analysis was performed to determine the most appropriate cut-off values. Univariate and multivariate logistic regression analyses were conducted to select potentially useful characteristics for predicting lymphatic leakage. Then, the area under the receiver operating characteristic (AUCROC) curves of the model were calculated. Kaplan-Meier analysis was used to draw and compare the two groups of survival curves. *P* < 0.05 was considered statistically significant.

## Results

### Clinical information of children with NB

According to the inclusion and exclusion criteria, a total of 186 children with NB (92 males and 94 females) were enrolled in this study, with a median age of 40 (23, 65) months. 32 children (17.2%) were INRG stage L2 and 93 children (50%) were INRG stage M. 124 children (66.7%) received preoperative neoadjuvant chemotherapy. IDRFs were found in 117 children (62.9%). The tumors of 70 children crossed the midline. The median diameter of the tumor was 6 (4, 8.2) cm. The median number of resected lymphatic regions was 3 (2, 5).

In our study, 174 children underwent open surgery and retroperitoneal drainage tubes were placed. Laparoscopic surgery was performed in 12 children without drainage tube, and the maximum diameter of the tumors were ranged from 1.3 to 4.9 cm. Two lymphatic regions were removed in 2 children and one lymphatic region was removed in the other 10 children. Besides, the 12 children returned to a normal diet soon after operation and had no ascites.

According to the definition of lymphatic leakage, 32 children were diagnosed as lymphatic leakage in 229 cases of abdominal NB operation, with an incidence of 14% (32/229). 154 children were included in the non-lymphatic leakage group (Table 1 and S1). Table 2 shows the surgical and other postoperative complications of the two groups. No death, major hemorrhage or nerve injury occurred in all children during the operation. Renal ischemia (17 children, 9.1%) occurs most frequently, and resection of kidney or ureter rarely occurs. No death, bleeding occurred in all children after operation. Pneumonia (39 children, 21%) and hepatic dysfunction (69 children, 37.1%) occur most frequently. Hepatic dysfunction is defined as the levels of alanine aminotransferase and aspartate aminotransferase in blood after surgery have exceed twice than the upper limit of normal.

**Table 1:** Clinical characteristics of 186 children with NB

Characteristics		lymphatic leakage^1,2^	Non-lymphatic leakage	Results^3^	*p* Value
		(*n* = 32)	(*n* = 154)		
Gender	Male	16 (50)	76 (49.4)	0.004	0.974
	Female	16 (50)	78 (50.6)		
Age (months)		35 (22, 46)	42 (23, 70)	1.626	0.106
INRG stage	L1	2 (6.3)	51 (33.1)	9.43	0.024
	L2	7 (21.8)	25 (16.2)		
	M	21 (65.6)	72 (46.8)		
	MS	2 (6.3)	6 (3.9)		
INRG risk	Very low	2 (6.3)	55 (35.7)	12.545	0.006
	Low	2 (6.3)	15 (9.7)		
	Intermediate	5 (15.6)	12 (7.8)		
	High	22 (68.7)	71 (46.1)		
	Unknown	1 (3.1)	1 (0.7)		
Pathology	NB&GNBn	32 (100)	124 (80.5)	7.433	0.006
	GNBi&GN	0	30 (19.5)		
Preoperative chemotherapy	Yes	30 (93.7)	94 (61)	12.757	< 0.001
	No	2 (6.3)	60 (39)		
IDRFs	Yes	28 (87.5)	89 (57.8)	12.021	0.002
	No	4 (12.5)	65 (42.2)		
Tumor crosses the midline at surgery	Yes	23 (71.9)	47 (30.5)	19.306	< 0.001
	No	9 (28.1)	107 (69.5)		
Maximum diameter of tumor at surgery (cm)		6.7 (5, 8.1)	5.9 (3.7, 8.2)	-1.334	0.184
Number of invaded blood vessels		4 (2, 6)	0 (0, 3)	4.977	< 0.001
Number of resected lymphatic regions		5 (4, 9)	3 (2, 4)	5.996	< 0.001
Middle-upper region resected	Yes	22 (68.8)	115 (74.7)	22.669	< 0.001
	No	10 (31.2)	39 (25.3)		
Degree of surgical resection	Gross total resection	32 (100)	154 (100)		
	< 90% resection	0	0		
Blood loss (ml)		30 (18.5, 50)	10 (5, 20)	4.345	< 0.0001
Operation time (h)		6.4 (4.9, 7.5)	3.5 (3, 5)	-5.777	< 0.0001

INRG, International Neuroblastoma Risk Group; IDRFs, Image-defined risk factors; NB, neuroblastoma; GNBn, ganglioneuroblastoma, nodular; GNBi, ganglioneuroblastoma, intermixed; GN, ganglioneuroma.

^1^Continuous variables are presented as the median and interquartile range.

^2^Classification variables are presented as numbers (percent).

^3^Results represent the z value of the Mann-Whitney test and the χ2 value of the chi-square test, respectively.

**Table 2:** Surgical and other postoperative complications of 186 children with NB

Complications		lymphatic leakage^1^	Non-lymphatic leakage	Total (*n* = 186)
		(*n* = 32)	(*n* = 154)	
Surgical complications	Intraoperative hypertension	2 (6.3)	1 (0.6)	3 (1.6)
	Diaphragm injury	1 (3.1)	5 (3.2)	6 (3.2)
	Renal/urologic injuries	4 (12.5)	18 (11.7)	22 (11.8)
	Vascular injury	1 (3.1)	2 (1.3)	3 (1.6)
Postoperative complications	Cardiovascular diseases	1 (3.1)	2 (1.3)	3 (1.6)
	Respiratory diseases	16 (50)	29 (18.8)	45 (24.2)
	Digestive diseases	14 (43.8)	58 (37.7)	72 (38.7)
	Adrenal/urologic diseases	7 (21.9)	12 (7.8)	19 (10.2)

^1^Classification variables are presented as numbers (percent).

### Clinical characteristics of lymphatic leakage

In the lymphatic leakage group, the median time for diagnosis of lymphatic leakage is 5 (4, 7) days after operation. The median maximum drain output was 405 (218.8, 542.5) ml/day, ranging from 60 ml/day to 2063 ml/day. The median time of abdominal drainage was 15 (10, 23) days, ranging from 8 to 42 days. The median hospital stay was 18 (13, 27) days, ranging from 8 to 53 days. There were 17 children (53%) with related complications due to lymphatic leakage, including 6 cases of hyponatremia, 4 cases of hypokalemia and 12 cases of hypoalbuminemia. All of 32 children underwent postoperative chemotherapy and the median time from operation to postoperative chemotherapy was 23 (17, 30) days, ranging from 14 to 74 days, of which 17 children (53.1%) were more than 3 weeks.

Compared with the non-lymphatic leakage group, the operation time, blood loss and postoperative drain output were significantly increased in the lymphatic leakage group (Table 3). And lymphatic leakage significantly increased the postoperative complications, prolonged the time of abdominal drainage and hospitalization, and delayed the postoperative chemotherapy (*p* < 0.05).

**Table 3:** Impact of lymphatic leakage on children with NB

Characteristics		lymphatic leakage^1,2^	Non-lymphatic leakage	Results^3^	*p* Value
		(*n* = 32)	(*n* = 154)		
Maximum abdominal drainage (ml/day)		405 (218.8, 542.5)	90 (40, 160)	6.937	< 0.001
Average abdominal drainage (ml/day)		138.4 (94.3, 211)	39.7 (25, 69.2)	6.841	< 0.001
Abdominal drainage time (days)		15 (10, 23)	8 (7, 10)	7.171	< 0.001
Complications related to lymphatic leakage		17 (53.1)	0	52.316	< 0.001
Hospitalization (days)		18 (13, 27)	11 (9, 13)	5.631	< 0.001
Time from operation to chemotherapy (days)^4^		23 (17, 30)	15 (13, 17)	-3.166	0.002
	> 3 weeks	17 (53.1)	18 (17.6)		< 0.0001
	< 3 weeks	15 (46.9)	84 (82.4)		
Three-year	OS	77.4% (95% CI: 64.0–93.6%)	85.5% (95% CI: 80.1–91.3%)		0.21
	EFS	58.6% (95% CI: 43.2–79.6%)	75.1% (95% CI: 68.5–82.4%)		0.057
	CILP	16.7% (95% CI: 2.3–29.0%)	4.9% (95% CI: 1.3–8.4%)		0.015

^1^Continuous variables are presented as the median and interquartile range.

^2^Classification variables are presented as numbers (percent).

^3^Results represent the z value of the Mann-Whitney test and the χ2 value of the chi-square test, respectively.

^4^Postoperative chemotherapy was performed in 32 cases in lymphatic leakage group and 102 cases in non-lymphatic leakage group.

**Fig. 3:**
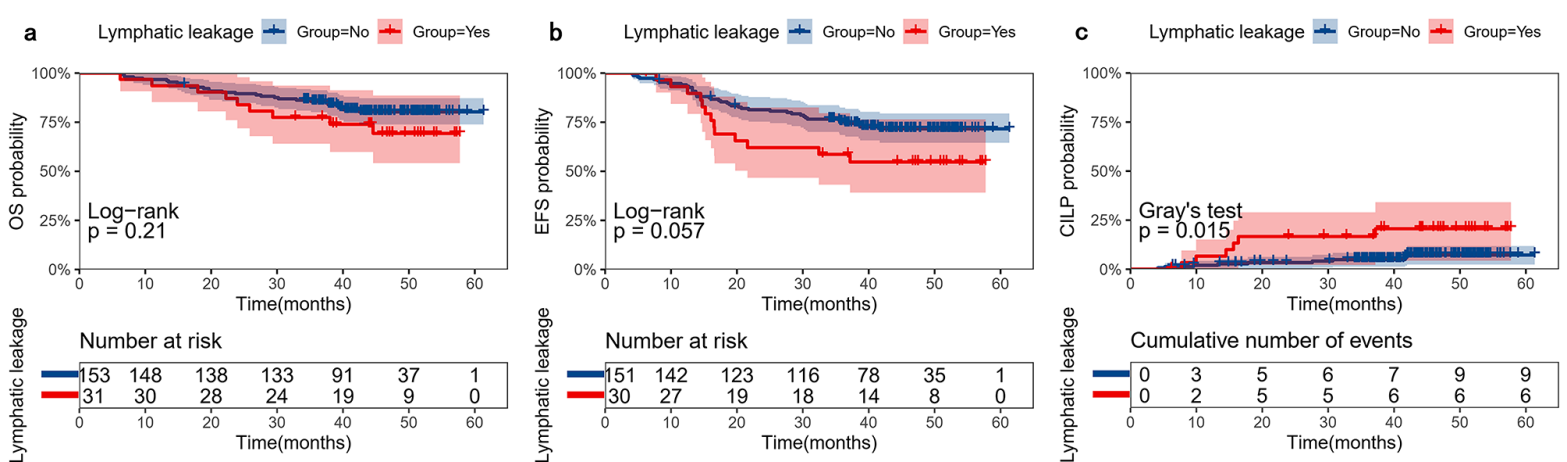
Prognosis of children with and without lymphatic leakage. (**a**) Overall survival (OS). (**b**) Event-free survival (EFS). (**c**) Cumulative incidence of local progression (CILP)

Three-year OS, EFS, and CILP for children with lymphatic leakage were 77.4% (95% CI: 64.0–93.6%), 58.6% (95% CI: 43.2–79.6%), and 16.7% (95% CI: 2.3–29.0%), respectively. Three-year OS, EFS, and CILP for children without lymphatic leakage were 85.5% (95% CI: 80.1–91.3%), 75.1% (95% CI: 68.5–82.4%), and 4.9% (95% CI: 1.3–8.4%), respectively. Compared with the non-lymphatic leakage group, there was no statistical difference in OS and EFS of lymphatic leakage group, with *p* values of 0.21 and 0.057 respectively (Fig. 3a and b). And the CILP of lymphatic leakage group increased significantly, with *p* values of 0.015 (Fig. 3c).

### Treatment of lymphatic leakage

**Fig. 4:**
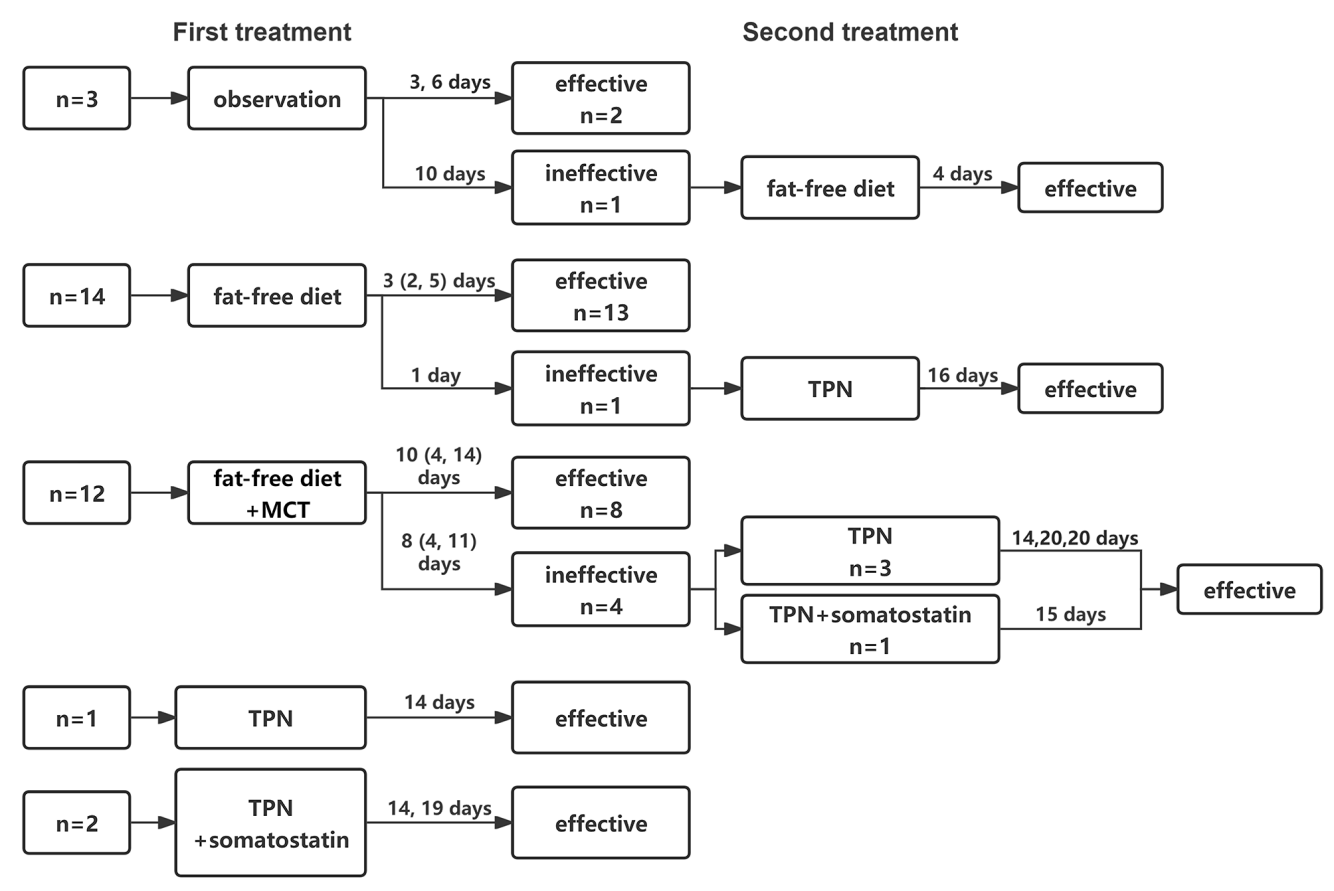
Different management of lymphatic leakage

In this study, 32 children with lymphatic leakage were cured by conservative treatment. None of the children had a large amount of ascites or progressive encapsulated effusion after removal of abdominal drainage. We applied a variety of treatments (Fig. 4).

3 children received observation after diagnosis. 2 of them were effective, and the drain output at diagnosis was 60 ml/day (3.8 ml/kg/day) and 30 ml/day (5 ml/kg/day), respectively. The other one child was ineffective and the drain output at diagnosis was 100 ml/day (6.1 ml/kg/day). The drain output was reduced after receiving fat-free diet.

14 children received fat-free diet after diagnosis. Among them, 13 children were effective, and the drain output at diagnosis was 110 (55, 167.5) ml/day (10 (4.4, 14.5) ml/kg/day). The other one child was ineffective and the drain output at diagnosis was 525 ml/day (42 ml/kg/day). The drain output decreased after accepting TPN.

12 children received fat-free diet with MCT after diagnosis. Among them, 8 children were effective, and the drain output at diagnosis was 155 (70, 175) ml/day (8.9(3, 15.2) ml/kg/day). The other 4 children were ineffective, and the drain output at diagnosis was 332.5 (86.3, 605) ml/day (25.7 (6.8, 40.9) ml/kg/day). The drain output decreased after accepting TPN with or without somatostatin.

1 child received TPN after diagnosis and the treatment was effective. The drain output at diagnosis was 860 ml/day (53.8 ml/kg/day). 2 children received TPN with somatostatin after diagnosis and the treatment was effective. The drain output at diagnosis was 530 ml/day (28.6 ml/kg/day) and 370 ml/day (49.3 ml/kg/day), respectively.

According to the above data, only some of the children with large drain output at diagnosis need to be treated with TPN. But the treatment of TPN always takes a long time, and the patients are prohibited from eating or drinking by mouth. In order to find the indication for use of TPN at diagnosis and reduce other ineffective treatments, we analyze the data from the TPN group and non-TPN group.

24 children were cured by non-TPN treatment (observation and fat-free diet) with the median treatment time of 4 (3, 9) days. The median drain output at diagnosis was 8.4 (4.7, 11.9) ml/kg/day. The other 8 children were cured by TPN with the median treatment time of 16 (14, 20) days. The median drain output at diagnosis was 37.8 (25.8, 44.8) ml/kg/day. of which 3 children were treated with somatostatin at the same time.

Comparing the drain output at diagnosis between non-TPN group and TPN group, we found that there were significant differences (*p* < 0.001). The cut-off value of the drain output calculated from the ROC curve is 17.2 ml/kg/day.

### Follow-up and prognosis

In our study, the last follow time was September 21, 2022, the median follow-up time was 46 (95% CI: 44–48) months, ranging from 6 to 61 months, and the prognosis of 186 children with NB was obtained through outpatient reexamination and/or telephone contact. Most patients were reviewed once a year or six months, telephone follow-up was once a year. The follow-up data of 7 children were partially missing. In this cohort, 147 children survived, of which 23 had tumor recurrence (5 children recurred in the surgical area). 37 children died, of which 32 had tumor recurrence (9 children recurred in the operation area).

In order to determine whether lymphatic leakage is an independent risk factor affecting the prognosis of children with NB, we further analyzed the risk factors affecting OS, EFS and CILP. The results showed that the independent risk factors of OS and EFS were INRG risk rather than lymphatic leakage, and the independent risk factors of CILP were maximum diameter of tumor at surgery and the number of invaded blood vessels rather than lymphatic leakage (Table S2-7). Therefore, the results of multivariate analysis showed that OS, EFS and CILP were not affected by lymphatic leakage.

### Risk factors of lymphatic leakage

Comparing the clinical characteristics of the two groups (Table 1), we found that there were significant differences in the INRG stage, INRG risk, IDRFs, tumor crossing the midline, preoperative chemotherapy, pathology, the number of invaded blood vessels, the number of resected lymphatic regions and middle-upper region resected.

According to the ROC curve, the cut-off values of the number of invaded blood vessels and the number of resected lymphatic regions were 4 vessels and 5 regions, respectively.

Through multivariate logistic regression analysis, we found that resection of 5 or more lymphatic regions was an independent risk factor for lymphatic leakage after NB surgery, with adjusted odds ratios (ORs) of 1.483 (1.122, 1.960) (Table 4). The AUC-ROC of the model was 0.833, and the sensitivity and specificity were 68.8% and 81.8%, respectively (Fig. S2).

**Table 4:** Multivariate logistic regression analysis of prediction of lymphatic leakage

Variables	Estimate	Standard error	Wald	*p* Value	OR (95% CI)
INRG stage	-0.266	0.479	0.308	0.579	0.767 (0.300, 1.960)
INRG risk	-0.039	0.349	0.012	0.911	0.962 (0.485, 1.907)
IDRFs	0.470	0.933	0.254	0.615	1.600 (0.257, 9.960)
Pathology	-18.820		0.000	0.998	0.000
Tumor crosses the midline	-0.023	0.633	0.001	0.972	1.023 (0.296, 3.535)
Preoperative chemotherapy	0.223	1.231	0.033	0.856	1.250 (0.112, 13.962)
Number of invaded blood vessels	0.031	0.106	0.084	0.772	1.031 (0.838, 1.268)
Number of resected lymphatic regions	0.394	0.142	7.654	0.006	1.483 (1.122, 1.960)
Middle-upper region resected	0.201	0.633	0.101	0.751	1.223 (0.354, 4.230)

INRG, International Neuroblastoma Risk Group; IDRFs, Image-defined risk factors.

## Discussion

At present, surgical treatment is still an important part of NB treatment. Primary tumors and metastatic lymph nodes in children with medium-and high-risk NB often wrap around important retroperitoneal vessels, so it is difficult to achieve 100% tumor resection. However, COG, SIOP and other studies [3–6] show that complete removal of tumors (no visible or palpable tumors), including involved lymph nodes, could improve local control, EFS or OS in high-risk children. Therefore, in recent years, regional lymph node dissection by vascular skeletalization has become a routine step in NB surgery. However, the increase of surgical trauma also led to an increase in postoperative lymphatic leakage.

Previous large studies have paid more attention to the impact of surgical resection on the survival of children with NB, and lack of detailed reports on surgical complications. The study of COG [3] reported surgical registration data of 110 children with high-risk NB and did not find major lymphatic leakage, but there was no detailed definition of the major chyle leaks and no specific description of the resection area. The study of SIOP [4] reported surgical registration data of 1531 children with high-risk NB. The major ascites was defined as a serious surgical complication, but there was no detailed definition and count of the major ascites, nor a specific description of the resection area. And these studies have a long-time range (more than 10 years) and include multiple medical centers and surgical teams, so there may be a lack of consistency in the description of lymphatic leakage. In recent years, there are many differences in the choice of treatment in some studies of lymphatic leakage after NB surgery. Froeba-Pohl [8] found that there was a trend toward shorter duration of lymphatic leakage if either no special therapy was chosen or TPN was administered. Qureshi [9] used fat-free/low-fat diet of MCT and Pio [11] used TPN with octreotide to treat lymphatic leakage. Therefore, we hope to provide more evidence for the treatment and prevention of lymphatic leakage (whether severe or mild) by reviewing the surgical data of our center.

In this study, 14% of the children had lymphatic leakage after operation, and the incidence of lymphatic leakage was similar to the results reported in the literature (9%∼40%) [7–11]. However, the incidence was significantly affected by diagnostic criteria.

Chylous drainage is an exact diagnostic criterion, but not all lymphatic leaks present as chylous. Anatomically, the cisterna chyli receives lymph from the intestinal trunk and the lumbar trunk, both of which are located in the area around the superior mesenteric artery, that is, the middle-upper region defined in this study (Fig. 1). If the intestinal trunk or cisterna chyli is injured, the drainage will become chylous after ingesting food with fat; if the lumbar trunk is injured, clear drainage could be leaked even if food with fat is ingested. In this situation, laboratory tests of the drainage fluid (such as triglycerides and cholesterol [20]) are needed to help diagnose and differentiate from major ascites of other causes (such as capillary leak syndrome [21, 22]). Lymphatic leakage cannot be diagnosed only according to the amount of drainage.

In the excluded cases, we also found 14 children of non-chylous major ascites, but unfortunately there were no relevant laboratory results (chylomicrons, triglycerids, cholesterol or lymphocyts count values), so the diagnosis could not be confirmed (Fig. S1). Although two studies on lymphatic leakage after NB surgery define major abdominal drainage (greater than 300 ml/day and 400 ml/day) as lymphatic leakage [8, 10], there may be significant differences in the amount of drainage among children with different body weight. Therefore, this diagnostic criterion may not be suitable for all children. In the absence of evidence, it is unreasonable to diagnose lymphatic leakage only according to the amount of drainage. In our center, due to our lack of attention to lymphatic leakage in the past, many children of lymphatic leakage and major ascites lacked biological values about drainage. Therefore, chylous fluid is still used as the diagnostic standard in this study. However, chylous fluid comes from the subjective judgment of doctors. For lymphatic leakage which chylous color is not obvious, the lack of biological values will reduce the detection rate. Therefore, in the follow-up clinical work and research, the examination of biological values of drainage fluid should be actively improved for major abdominal drainage with or without chylus.

In addition, systematic placement of abdominal drainage tubes during laparotomy may also increase the detection rate of lymphatic leakage, especially in children with low drain output. In this study, it was also found that 2 children with low drainage could be cured without any treatment. In addition, no postoperative peritoneal effusion was found in 12 children who underwent laparoscopic surgery. Physiologically, the peritoneum has the ability to absorb a small amount of ascites. Therefore, for laparotomy with only 2 or less lymphatic regions removed, it may also be safe not to place a drainage tube, thus reducing the over-detection of lymphatic leakage. However, drainage tubes are recommended for children who received more lymphatic regions resection, especially those who received 5 or more lymphatic regions resection.

The lymphatic system is not only an important part of humoral circulation, but also involved in nutrient metabolism and immune function. Lymphatic leakage can directly cause the loss of nutrients, and even lead to hypovolemic shock [7, 11, 23]. Our results also reflect this characteristic of lymphatic leakage. Although there was no shock after active treatment, 53% of the children with lymphatic leakage still had hypoproteinemia and electrolyte disorder. Moreover, lymphatic leakage needs extra time to treat, resulting in prolonged time of abdominal drainage and hospitalization, which is very disadvantageous to children with NB who need chemotherapy after operation. Chemotherapy is usually carried out 3 weeks after operation [8]. This study found that 53.1% of the children with lymphatic leakage failed to start chemotherapy in time, which may affect the overall therapeutic effect.

Summarize the follow-up data of patients, we found that the 3-year OS and EFS in the lymphatic leakage group were lower than those in the non-lymphatic leakage group, while 3-year CILP was higher in lymphatic leakage group, which we consider to be affected by confounding factors. Subsequently, we analyzed the risk factors affecting OS, EFS and CILP. The results showed that lymphatic leakage was neither an independent risk factor for OS, nor for EFS and CILP. Therefore, lymphatic leakage does not affect the prognosis of children with NB. Although lymphatic leakage is not as dangerous as bleeding and pancreatic fistula, long-term abdominal drainage will still adversely affect postoperative complications and follow-up treatment. It has been emphasized in previous articles [8–10] that chemotherapy or other systemic treatment of NB children with lymphatic leakage should not be delayed. Although there is a lack of clear recommended time for adjuvant chemotherapy after NB surgery, many studies have suggested that postoperative adjuvant chemotherapy should be carried out as early as possible [24, 25]. Emberesh [26] reported that there was a delay of systematic treatment (including chemotherapy, radiotherapy and immunotherapy) in 2 children with high-risk NB. Even if they subsequently received targeted immunotherapy, they did not escape the recurrence and progression of tumor. In addition, Hillier [27] found that the delay of induction chemotherapy is a risk factor for engraftment of platelet and hemoglobin in children with NB after autologous stem cell transplantation. The extension of engraftment time will increase the risk of infection and malnutrition, and then affect the safety and efficacy of autologous stem cell transplantation. Therefore, we should pay attention to lymphatic leakage and take active treatment measures to reduce or avoid delayed chemotherapy. In our study, few patients received chemotherapy with a drainage tube before, but now it is being tried.

At present, conservative treatment is the main treatment of lymphatic leakage after NB surgery [8, 9, 11]. In our study, children with lymphatic leakage were cured by different conservative treatments. Although fat-free diet and TPN are reported in the literature to be effective in the treatments of lymphatic leakage [7–10, 28], the range of lymphatic leakage is very large, and the severity is also different. Previous studies have not recommended treatment according to the drain output at diagnosis of lymphatic leakage [7–9, 11, 12, 29–31].

In our study, we found that children with lymphatic leakage less than 17.2 ml/kg/day at diagnosis can usually be cured by a fat-free diet, and the treatment time is short. Treatments for children with lymphatic leakage larger than 17.2 ml/kg/day at diagnosis are not effective if they are treated with fat-free diet alone, and they usually need to be cured by TPN with 2 weeks or more.

Therefore, we believe that conservative treatment is still the first-line for lymphatic leakage. For children with lymphatic leakage whose drain output at diagnosis is less than 17.2 ml/kg/day, we recommend a fat-free diet. Although there is a possibility of self-recovery (2 children did not change their diet after the appearance of chylus), we do not encourage “wait and observe” in view of the possible prolongation of treatment time. For the children whose drain output at diagnosis is larger than 17.2 ml/kg/day, the early application of TPN is recommended. At the same time, we should also pay attention to the occurrence of electrolyte disturbance and hypoproteinemia and supplement nutrition in time. And keep the drainage unobstructed to avoid a large peritoneal effusion oppressing the surrounding organs.

It has been reported that MCT and somatostatin also have therapeutic effects on lymphatic leakage [7, 9, 10], but in this study, the number of children treated with MCT and somatostatin is small and no effective conclusion could be drawn.

Due to the limitations of the retrospective study, there is no consistent protocol before treatment and difficult to conclude on when shall we move from one management to another. Therefore, it is necessary to carry out a prospective study with a clear protocol to draw reliable conclusions.

In order to treat refractory lymphatic leakage more quickly, some researchers have also applied minimally invasive treatment based on lymphangiography, including endolymphatic exclusion, intranodal lipiodol injection and lymphatic embolization [12, 30, 31]. Although the results are limited to case reports and small case series, the overall effect is satisfactory. Due to the low incidence of refractory lymphatic leakage after solid tumor resection in children, the research on minimally invasive treatment is still very limited. But it is indeed an alternative to surgery. Other surgical treatments have been reported in previous literature, such as chylous fistula repair, and peritoneovenous shunting [7, 10, 32–35], but they are all case reports and the surgical indications are controversial.

Finally, in order to prevent the occurrence of lymphatic leakage, the clinical characteristics of the lymphatic leakage group and the non-lymphatic leakage group were compared. We found that resection of 5 or more lymphatic regions was an independent risk factor. This is consistent with the anatomy of the retroperitoneal lymphatic system, and the more lymphatic regions are removed, the higher the risk of lymphatic vessels injury. It is also reported in the literature that the number of lymph nodes resected is closely related to lymphatic leakage [9], but because the number of lymph nodes is obtained through postoperative pathological results, it is often later than the occurrence of lymphatic leakage. Therefore, the number of resected lymphatic regions is more predictable and operable, which can move the preventive measures forward. For children with possible lymphatic leakage, we should actively look for and repair the injured lymphatic vessels during the operation and pay more attention to it after the operation and treat it as soon as possible.

Chu [10] also suggests that prophylactic mesenteric lymphatic ligation can achieve good results, and the use of a high-fat diet before operation can produce chylous lymphangiographic effect, which is helpful for the operator to identify lymphatic vessels. At present, in addition to a high-fat diet, the intraoperative application of nano-carbon and indocyanine green can also achieve the visualization of lymphatic vessels [36–38]. With the continuous progress in the technology of identifying lymphatic vessels, we believe that it can reduce the occurrence of lymphatic leakage in the future.

Compared with previous literature reports [8–11], the advantage of this study is that the research time is more concentrated, and we avoid affecting the research results due to the differences in surgical techniques, surgical methods and perioperative management in different periods. However, this study is a single-center retrospective study. Although the diagnosis, treatment and risk factors of lymphatic leakage after NB surgery were analyzed in detail, the conclusions still need to be verified by a large sample of prospective studies.

## Conclusion

Lymphatic leakage is a common postoperative complication of NB surgery. It does not an independent risk factor affecting the prognosis of children, but long-term loss of lymph fluid will lead to complications and delay the time of treatment. Therefore, early diagnosis and treatment are needed for lymphatic leakage. For children with lymphatic leakage whose drain output at diagnosis is less than 17.2 ml/kg/day, a fat-free diet is recommended first. Early application of TPN is recommended for those who have drain output at diagnosis of greater than 17.2 ml/kg/day. Resection of 5 or more lymphatic regions is an independent risk factor for lymphatic leakage. More attention should be paid to children who are likely to have lymphatic leakage. In order to reduce the occurrence of lymphatic leakage, it should be actively found and ligated after resection of the tumor.

### Electronic supplementary material

Below is the link to the electronic supplementary material.

Supplementary Material 1

Supplementary Material 2

Supplementary Material 3

## Data Availability

The datasets generated during and/or analyzed during the current study are available from the corresponding author on reasonable request.

## Abbreviations

NB: Neuroblastoma
TPN: Total parenteral nutrition
INRG: International Neuroblastoma Risk Group
IDRFs: Image-defined risk factors
OS: Overall survival
EFS: Event-free survival
CILP: Cumulative incidence of local progression
MCT: Medium chain triglycerides
ROC: Receiver operating characteristic
AUCROC: Area under the receiver operating characteristic
IVC: Inferior vena cava
SMA: Superior mesenteric artery
IMA: Inferior mesenteric artery
