# Supplementary material for: Risk factors, impact and treatment of postoperative lymphatic leakage in children with abdominal neuroblastoma operated on by laparotomy
Source: BMC Surg. 2024 May 29;24:168. doi: 10.1186/s12893-024-02459-3 (PMC11134958; doi:10.1186/s12893-024-02459-3)
Supplement: Supplementary file 1 — Supplementary Material 1 [file 12893_2024_2459_MOESM1_ESM.docx]

| Table S1. Supplementary clinical information of 186 children with NB | | | | | |
| --- | --- | --- | --- | --- | --- |
| Characteristics | | lymphatic leakage^1^ | Non-lymphatic leakage | Results^2^ | *p* Value |
|  |  | (n=32) | (n=154) |  |  |
| Primary site | Adrenal | 26 (81.3) | 114 (74) | 0.743 | 0.389 |
|  | Retroperitoneum | 6 (18.7) | 40 (26) |  |  |
| Primary site of origin | Left | 13 (40.6) | 68 (44.2) | 1.936 | 0.586 |
|  | Right | 16 (50) | 79 (51.3) |  |  |
|  | Middle | 1 (3.1) | 4 (2.6) |  |  |
|  | Bilateral | 2 (6.3) | 3 (1.9) |  |  |
| Grade of neuroblastic differentiation | Differentiating | 6 (18.7) | 38 (24.7) | 2.89 | 0.089 |
|  | Poorly differentiated | 25 (78.2) | 69 (44.8) |  |  |
|  | Unknown | 1 (3.1) | 47 (30.5) |  |  |
| *MYCN* | Normal | 16 (50) | 96 (62.4) | 3.09 | 0.213 |
|  | Gain | 9 (28.1) | 33 (21.4) |  |  |
|  | Amplified | 7 (21.9) | 18 (11.7) |  |  |
|  | Unknown | 0 | 7 (4.5) |  |  |
| *1p36* | Normal | 20 (62.6) | 98 (63.6) | 1.51 | 0.68 |
|  | Imbalance | 5 (15.6) | 23 (15) |  |  |
|  | Loss | 5 (15.6) | 25 (16.2) |  |  |
|  | Amplified | 1 (3.1) | 1 (0.7) |  |  |
|  | Unknown | 1 (3.1) | 7 (4.5) |  |  |
| *11q23* | Normal | 19 (59.4) | 89 (57.8) | 1.815 | 0.612 |
|  | Imbalance | 5 (15.6) | 14 (9.1) |  |  |
|  | Loss | 7 (21.9) | 39 (25.3) |  |  |
|  | Amplified | 0 | 3 (2) |  |  |
|  | Unknown | 1 (3.1) | 9 (5.8) |  |  |
| ^1^Classification variables are presented as numbers (percent). | | | | | |
| ^2^Results represent the z value of the Mann-Whitney test and the χ2 value of the chi-square test, respectively. | | | | | |

| Table S2. Clinical characteristics of overall survival in 186 children with NB. | | | | | |
| --- | --- | --- | --- | --- | --- |
| Characteristics | | Alive^1,2^ (n=147) | Death (n=37) | Results^3^ | *p* Value |
| lymphatic leakage | Yes | 125 (85.0) | 9 (24.3) | 1.848 | 0.174 |
|  | No | 22 (15.0) | 28 (75.7) |  |  |
| Gender | Male | 73 (49.7) | 18 (48.6) | 0.012 | 0.912 |
|  | Female | 74 (50.3) | 19 (51.4) |  |  |
| Age (months) |  | 38 (20, 67) | 43 (28, 61) | 0.110 | 0.912 |
| Primary site | Adrenal | 108 (73.5) | 30 (81.1) | 0.913 | 0.339 |
|  | Retroperitoneum | 39 (26.5) | 7 (18.9) |  |  |
| Primary site of origin | Left | 59 (40.1) | 20 (54.1) | 4.927 | 0.177 |
|  | Right | 80 (54.4) | 15 (40.5) |  |  |
|  | Middle | 5 (3.4) | 0 |  |  |
|  | Bilateral | 3(2.1) | 2 (5.4) |  |  |
| INRG risk | Very low | 57 (38.8) | 0 | 33.878 | **<0.001** |
|  | Low | 17 (11.6) | 0 |  |  |
|  | Intermediate | 15 (10.2) | 3 (8.1) |  |  |
|  | High | 57 (38.8) | 33 (89.2) |  |  |
|  | Unknown | 1 (0.6) | 1 (2.7) |  |  |
| IDRFs | Yes | 83 (56.5) | 32 (86.5) | 11.369 | **0.001** |
|  | No | 64 (43.5) | 5 (13.5) |  |  |
| Tumor crosses the midline | Yes | 49 (33.3) | 21 (56.8) | 6.881 | **0.009** |
|  | No | 98 (66.7) | 16 (43.2) |  |  |
| Maximum diameter of tumor at surgery (cm) |  | 5.8 (3.7, 7.9) | 7.3 (5.0, 11.0) | -2.193 | **0.030** |
| Number of invaded blood vessels |  | 0 (0, 3) | 2 (1, 5) | 3.119 | **0.002** |
| Pathology | NB | 79 (53.7) | 22 (59.5) | 9.942 | **0.019** |
|  | GNBn | 38 (28.9) | 15 (40.5) |  |  |
|  | GNBi | 19 (12.9) | 0 |  |  |
|  | GN | 11 (7.5) | 0 |  |  |
| *MYCN* | Normal | 93 (63.3) | 18 (48.7) | 7.740 | **0.021** |
|  | Gain | 33 (22.4) | 9 (24.3) |  |  |
|  | Amplified | 14 (9.5) | 10 (27.0) |  |  |
|  | Unknown | 7 (4.8) | 0 |  |  |
| *1p36* | Normal | 101 (68.7) | 17 (46.0) | 8.428 | **0.038** |
|  | Imbalance | 19 (12.9) | 9 (24.3) |  |  |
|  | Loss | 19 (12.9 | 9 (24.3) |  |  |
|  | Amplified | 1 (0.7) | 1 (2.7) |  |  |
|  | Unknown | 7 (4.8) | 1 2.7) |  |  |
| *11q23* | Normal | 89 (60.5) | 17 (46.0) | 4.354 | 0.226 |
|  | Imbalance | 15 (10.2) | 4 (10.8) |  |  |
|  | Loss | 32 (21.8) | 14 (37.8) |  |  |
|  | Amplified | 2 (1.4) | 1 (2.7) |  |  |
|  | Unknown | 9 (6.1) | 1 (2.7) |  |  |
| Degree of surgical resection | Gross total resection | 147 (100) | 37 (100) |  |  |
|  | < 90% resection | 0 | 0 |  |  |
| Abdominal drainage time (days) |  | 8 (7, 10) | 10 (8, 14) | -2.860 | **0.005** |
| Hospitalization (days) |  | 11 (9, 14) | 14 (12, 17) | -2.497 | **0.013** |
| Time from operation to chemotherapy (days) |  | 15 (14, 23) | 15 (14, 18) | -0.773 | 0.439 |
| INRG, International Neuroblastoma Risk Group; IDRFs, Image-defined risk factors; NB, neuroblastoma; GNBn, ganglioneuroblastoma, nodular;  GNBi, ganglioneuroblastoma, intermixed; GN, ganglioneuroma. | | | | | |
| ^1^Continuous variables are presented as the median and interquartile range. | | | | | |
| ^2^Classification variables are presented as numbers (percent). | | | | | |
| ^3^Results represent the z(t) value of the Mann-Whitney test and the χ2 value of the chi-square test, respectively. | | | | | |

| Table S3. Multivariate logistic regression analysis of overall survival in 186 children with NB. | | | | | |
| --- | --- | --- | --- | --- | --- |
| Variables | Estimate | Standard error | Wald | *p* Value | OR (95% CI) |
| INRG risk | 1.835 | 0.565 | 10.543 | **0.001** | 6.268 (2.070, 18.980) |
| IDRFs | 0.609 | 0.716 | 0.724 | 0.395 | 1.839 (0.452, 7.479) |
| Tumor crosses the midline | -0.921 | 0.622 | 2.192 | 0.139 | 0.398 (0.118, 1.348) |
| Number of invaded blood vessels | 0.092 | 0.090 | 1.031 | 0.310 | 1.096 (0.918, 1.308) |
| *MYCN* | 0.208 | 0.317 | 0.429 | 0.513 | 1.231 (0.661, 2.293) |
| Pathology | 0.269 | 0.463 | 0.337 | 0.561 | 1.309 (0.528, 3.246) |
| Abdominal drainage time (days) | 0.002 | 0.086 | 0.000 | 0.986 | 1.002 (0.846, 1.185) |
| Hospitalization (days) | 0.012 | 0.081 | 0.022 | 0.881 | 1.012 (0.864, 1.185) |
| *1p36* | 0.081 | 0.287 | 0.079 | 0.779 | 1.084 (0.617, 1.903) |
| Maximum diameter of tumor at surgery (cm) | 0.158 | 0.080 | 3.855 | 0.050 | 1.171 (1.000, 1.370) |
| INRG, International Neuroblastoma Risk Group; IDRFs, Image-defined risk factors. | | | | | |

| Table S4. Clinical characteristics of event-free survival in 186 children with NB. | | | | | |
| --- | --- | --- | --- | --- | --- |
| Characteristics | | Event ^1,2^ (n=59) | No Event (n=124) | Results^3^ | *p* Value |
| lymphatic leakage | Yes | 15 (25.4) | 16 (12.9) | 4.454 | **0.035** |
|  | No | 44 (74.6) | 108 (87.1) |  |  |
| Gender | Male | 28 (47.5) | 63 (50.8) | 0.179 | 0.672 |
|  | Female | 31 (52.5) | 61 (49.2) |  |  |
| Age (months) |  | 50 (37, 71) | 34 (17, 55) | -1.782 | 0.076 |
| Primary site | Adrenal | 50 (84.7) | 87 (70.2) | 4.519 | **0.034** |
|  | Retroperitoneum | 9 (15.3) | 37 (29.8) |  |  |
| Primary site of origin | Left | 30 (50.8) | 49 (39.5) | 2.573 | 0.462 |
|  | Right | 26 (44.1) | 68 (54.9) |  |  |
|  | Middle | 1 (1.7) | 4 (3.2) |  |  |
|  | Bilateral | 2 (3.4) | 3 (2.4) |  |  |
| INRG risk | Very low | 0 | 57 (46.0) | 67.761 | **<0.001** |
|  | Low | 0 | 17 (13.7) |  |  |
|  | Intermediate | 4 (6.8) | 13 (10.5) |  |  |
|  | High | 54 (91.5) | 36 (29.0) |  |  |
|  | Unknown | 1 (1.7) | 1 (0.8) |  |  |
| IDRFs | Yes | 52 (88.1) | 62 (50.0) | 24.753 | **<0.001** |
|  | No | 7 (11.9) | 62 (50.0) |  |  |
| Tumor crosses the midline | Yes | 34 (57.6) | 35 (28.2) | 14.713 | **<0.001** |
|  | No | 25 (42.4 | 89 (71.8) |  |  |
| Maximum diameter of tumor at surgery (cm) |  | 6.9 (5.0, 10.1) | 5.6 (3.1, 7.8) | -2.165 | **0.032** |
| Number of invaded blood vessels |  | 2 (1, 5) | 0 (0, 3) | 4.439 | **<0.001** |
| Pathology | NB | 37 (62.7) | 64 (51.6) | 17.579 | **0.001** |
|  | GNBn | 22 (37.3) | 30 (24.2) |  |  |
|  | GNBi | 0 | 19 (15.3) |  |  |
|  | GN | 0 | 11 (8.9) |  |  |
| *MYCN* | Normal | 32 (54.2) | 80 (64.5) | 3.549 | 0.170 |
|  | Gain | 17 (28.8) | 25 (20.2) |  |  |
|  | Amplified | 10 (17.0) | 12 (9.7) |  |  |
|  | Unknown | 0 | 7 (5.6) |  |  |
| *1p36* | Normal | 31 (52.6) | 87 (70.2) | 7.918 | **0.048** |
|  | Imbalance | 14 (23.7) | 14 (11.3) |  |  |
|  | Loss | 12 (20.3) | 15 (12.1) |  |  |
|  | Amplified | 1 (1.7) | 1 (0.8) |  |  |
|  | Unknown | 1 (1.7) | 7 (5.6) |  |  |
| *11q23* | Normal | 23 (38.9) | 82 (66.1) | 19.533 | **<0.001** |
|  | Imbalance | 7 (11.9) | 12 (9.7) |  |  |
|  | Loss | 27 (45.8) | 19 (15.3) |  |  |
|  | Amplified | 1 (1.7) | 2 (1.6) |  |  |
|  | Unknown | 1 (1.7) | 9 (7.3) |  |  |
| Degree of surgical resection | Gross total resection | 59 (100) | 124 (100) |  |  |
|  | < 90% resection | 0 | 0 |  |  |
| Abdominal drainage time (days) |  | 10 (8, 14) | 8 (7, 10) | 4.497 | **<0.001** |
| Hospitalization (days) |  | 14 (11, 17) | 11 (9, 13) | 4.545 | **<0.001** |
| Time from operation to chemotherapy (days) |  | 15 (14, 17) | 16 (14, 24) | 0.031 | 0.975 |
| INRG, International Neuroblastoma Risk Group; IDRFs, Image-defined risk factors; NB, neuroblastoma; GNBn, ganglioneuroblastoma, nodular;  GNBi, ganglioneuroblastoma, intermixed; GN, ganglioneuroma. | | | | | |
| ^1^Continuous variables are presented as the median and interquartile range. | | | | | |
| ^2^Classification variables are presented as numbers (percent). | | | | | |
| ^3^Results represent the z(t) value of the Mann-Whitney test and the χ2 value of the chi-square test, respectively. | | | | | |

| Table S5. Multivariate logistic regression analysis of event-free survival in 186 children with NB. | | | | | |
| --- | --- | --- | --- | --- | --- |
| Variables | Estimate | Standard error | Wald | *p* Value | OR (95% CI) |
| INRG risk | 2.371 | 0.590 | 16.136 | **<0.001** | 10.708 (3.367, 34.052) |
| Lymphatic leakage after surgery | -0.676 | 0.709 | 0.908 | 0.341 | 0.509 (0.127, 2.043) |
| Primary site | 0.071 | 0.695 | 0.011 | 0.918 | 1.074 (0.275, 4.192) |
| IDRFs | 0.897 | 0.699 | 1.646 | 0.199 | 2.452 (0.623, 9.652) |
| Tumor crosses the midline | -0.439 | 0.609 | 0.519 | 0.471 | 0.645 (0.196, 2.127) |
| Number of invaded blood vessels | 0.019 | 0.094 | 0.039 | 0.844 | 1.019 (0.847, 1.225) |
| *11q23* | 0.344 | 0.153 | 1.845 | 0.174 | 1.410 (0.859, 2.315) |
| Pathology | -0.262 | 0.486 | 0.291 | 0.589 | 0.769 (0.297, 1.995) |
| Abdominal drainage time (days) | -0.003 | 0.092 | 0.001 | 0.971 | 0.997 (0.832, 1.194) |
| Hospitalization (days) | 0.064 | 0.080 | 0.626 | 0.429 | 1.066 (0.910, 1.247) |
| Maximum diameter of tumor at surgery (cm) | 0.125 | 0.085 | 2.153 | 0.142 | 1.133 (0.959, 1.339) |
| *1p36* | -0.247 | 0.281 | 0.774 | 0.379 | 0.781 (0.451, 1.354) |
| INRG, International Neuroblastoma Risk Group; IDRFs, Image-defined risk factors. | | | | | |

| Table S6. Clinical characteristics of CILP in 186 children with NB. | | | | | |
| --- | --- | --- | --- | --- | --- |
| Characteristics | | Event ^1,2^ (n=15) | No Event (n=166) | Results^3^ | *p* Value |
| lymphatic leakage | Yes | 6 (40.0) | 25 (15.1) | 6.029 | **0.014** |
|  | No | 9 (60.0) | 141 (84.9) |  |  |
| Gender | Male | 9 (60.0) | 80 (48.2) | 0.767 | 0.381 |
|  | Female | 6 (40.0) | 86 (51.8) |  |  |
| Age (months) |  | 42 (28, 69) | 40 (21, 63) | -1.011 | 0.313 |
| Primary site | Adrenal | 11 (73.3) | 124 (74.7) | 0.014 | 0.907 |
|  | Retroperitoneum | 4 (26.7) | 42 (25.3) |  |  |
| Primary site of origin | Left | 8 (53.3) | 70 (42.2) | 3.240 | 0.356 |
|  | Right | 5 (33.3) | 88 (53.0) |  |  |
|  | Middle | 1 (6.7) | 4 (2.4) |  |  |
|  | Bilateral | 1 (6.7) | 4 (2.4) |  |  |
| INRG risk | Very low | 0 | 57 (34.3) | 12.266 | **0.007** |
|  | Low | 0 | 17 (10.3) |  |  |
|  | Intermediate | 1 (6.7) | 16 (9.6) |  |  |
|  | High | 13 (86.6) | 75 (45.2) |  |  |
|  | Unknown | 1 (6.7) | 1 (0.6) |  |  |
| IDRFs | Yes | 12 (80.0) | 101 (60.8) | 2.152 | 0.142 |
|  | No | 3 (20.0) | 65 (39.2) |  |  |
| Tumor crosses the midline | Yes | 11 (73.3) | 58 (34.9) | 8.597 | **0.003** |
|  | No | 4 (26.7) | 108 (65.1) |  |  |
| Maximum diameter of tumor at surgery (cm) |  | 9.2 (5.3, 11.4) | 6 (4, 8) | -2.341 | **0.020** |
| Number of invaded blood vessels |  | 5 (2, 7) | 1 (0, 3) | 2.955 | **0.003** |
| Pathology | NB | 9 (60.0) | 91 (54.8) | 3.589 | 0.309 |
|  | GNBn | 6 (40.0) | 45 (27.1) |  |  |
|  | GNBi | 0 | 19 (11.5) |  |  |
|  | GN | 0 | 11 (6.6) |  |  |
| *MYCN* | Normal | 7 (46.7) | 103 (62.1) | 6.385 | **0.041** |
|  | Gain | 3 (20.0) | 39 (23.5) |  |  |
|  | Amplified | 5 (33.3) | 17 (10.2) |  |  |
|  | Unknown | 0 | 7 (4.2) |  |  |
| *1p36* | Normal | 7 (46.7) | 110 (66.2) | 2.847 | 0.416 |
|  | Imbalance | 4 (26.7) | 23 (13.9) |  |  |
|  | Loss | 3 (20.0) | 24 (14.5) |  |  |
|  | Amplified | 0 | 2 (1.2) |  |  |
|  | Unknown | 1 (6.6) | 7 (4.2) |  |  |
| *11q23* | Normal | 8 (53.3) | 97 (58.5) | 0.547 | 0.908 |
|  | Imbalance | 2 (13.3) | 16 (9.6) |  |  |
|  | Loss | 4 (26.7) | 41 (24.7) |  |  |
|  | Amplified | 0 | 3 (1.8) |  |  |
|  | Unknown | 1 (6.7) | 9 (5.4) |  |  |
| Degree of surgical resection | Gross total resection | 15 (100) | 166 (100) |  |  |
|  | < 90% resection | 0 | 0 |  |  |
| Abdominal drainage time (days) |  | 14 (9, 16) | 8 (7, 10) | -3.290 | **0.001** |
| Hospitalization (days) |  | 15 (12, 19) | 11 (10, 15) | -2.595 | **0.010** |
| Time from operation to chemotherapy (days) |  | 17 (15, 18) | 15 (14, 23) | 0.239 | 0.811 |
| INRG, International Neuroblastoma Risk Group; IDRFs, Image-defined risk factors; NB, neuroblastoma; GNBn, ganglioneuroblastoma, nodular; GNBi, ganglioneuroblastoma, intermixed; GN, ganglioneuroma. | | | | | |
| ^1^Continuous variables are presented as the median and interquartile range. | | | | | |
| ^2^Classification variables are presented as numbers (percent). | | | | | |
| ^3^Results represent the z(t) value of the Mann-Whitney test and the χ2 value of the chi-square test, respectively. | | | | | |

| Table S7. Multivariate logistic regression analysis of CILP in 186 children with NB. | | | | | |
| --- | --- | --- | --- | --- | --- |
| Variables | Estimate | Standard error | Wald | *p* Value | OR (95% CI) |
| INRG risk | 1.936 | 1.137 | 2.902 | 0.088 | 6.934 (0.747, 64.357) |
| Lymphatic leakage after surgery | -0.466 | 0.946 | 0.243 | 0.622 | 0.628 (0.098, 4.007) |
| Maximum diameter of tumor at surgery (cm) | 0.195 | 0.091 | 4.579 | **0.032** | 1.215 (1.017, 1.453) |
| Tumor crosses the midline | -0.340 | 0.843 | 0.162 | 0.687 | 0.712 (0.136, 3.716) |
| Number of invaded blood vessels | 0.197 | 0.095 | 4.341 | **0.037** | 1.218 (1.012, 1.467) |
| Abdominal drainage time (days) | 0.031 | 0.043 | 0.514 | 0.474 | 1.031 (0.948, 1.121) |
| Hospitalization (days) | -0.176 | 0.144 | 1.487 | 0.223 | 0.839 (0.632, 1.113) |
| *MYCN* | 0.634 | 0.386 | 2.703 | 0.100 | 1.885 (0.885, 4.013) |
| INRG, International Neuroblastoma Risk Group. | | | | | |
