# Supplementary figures and images for: Risk factors, impact and treatment of postoperative lymphatic leakage in children with abdominal neuroblastoma operated on by laparotomy
Source: BMC Surg. 2024 May 29;24:168. doi: 10.1186/s12893-024-02459-3 (PMC11134958; doi:10.1186/s12893-024-02459-3)

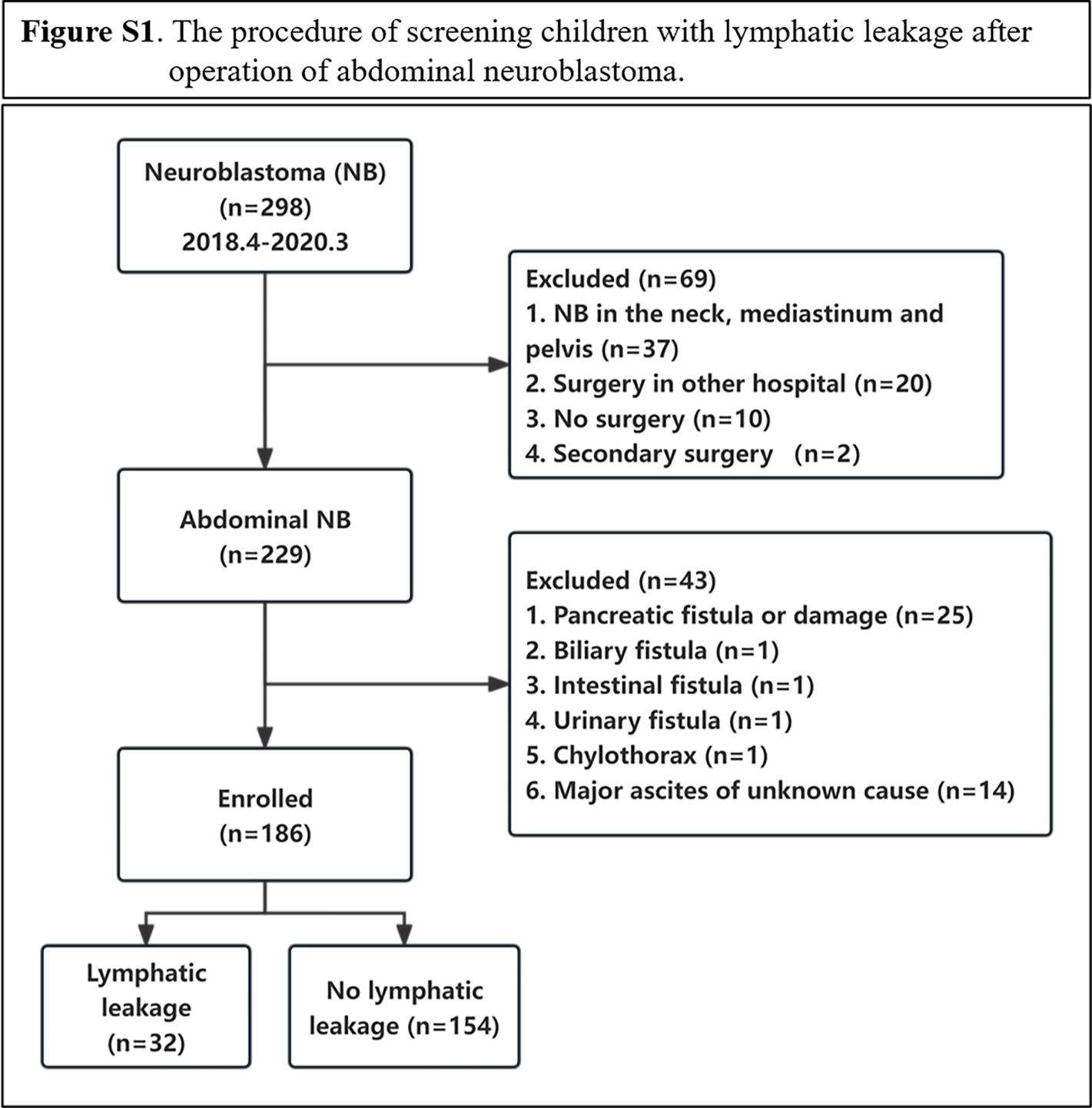

Supplement: Supplementary file 2 — Supplementary Material 2 [file 12893_2024_2459_MOESM2_ESM.docx]

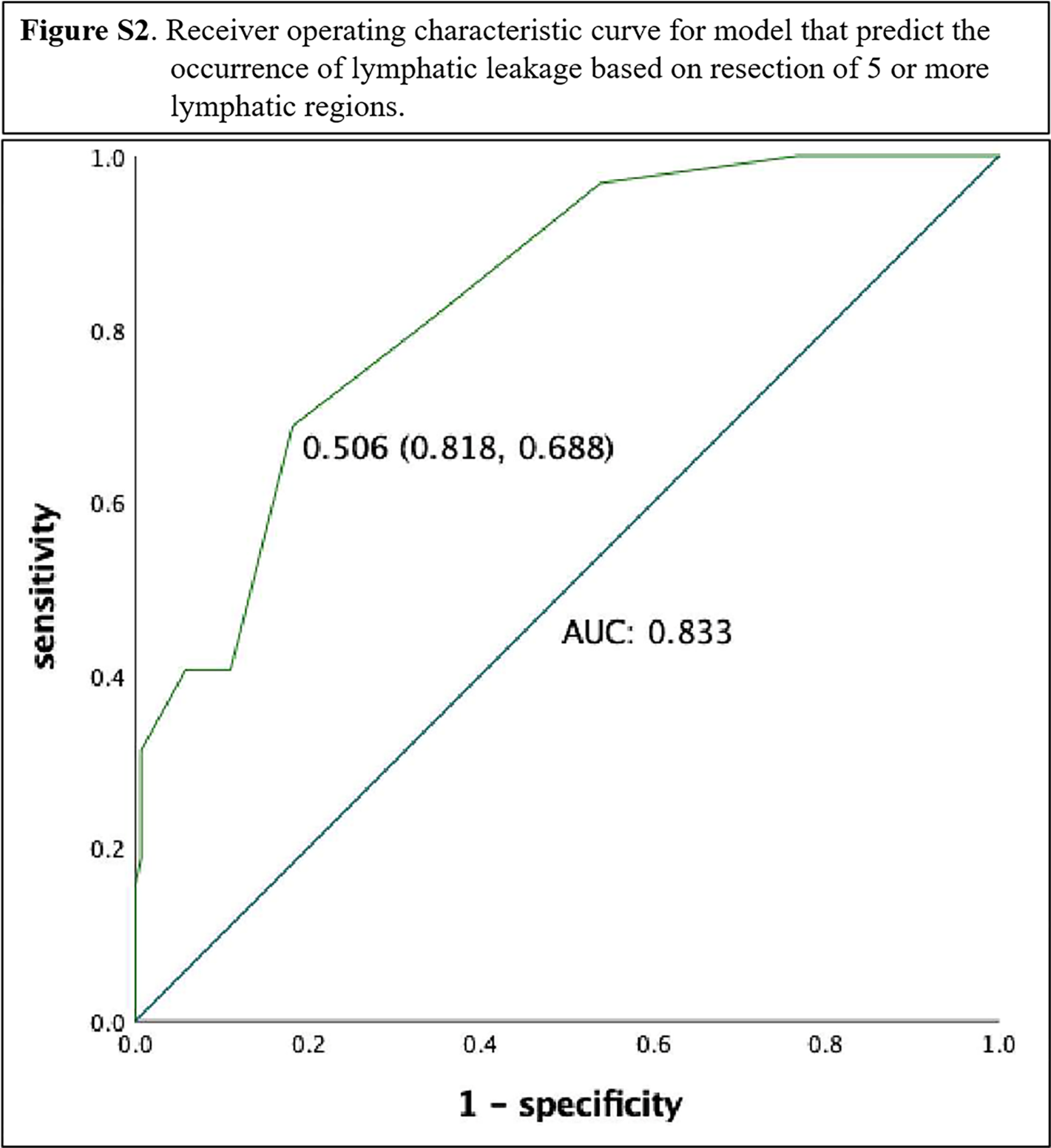

Supplement: Supplementary file 3 — Supplementary Material 3 [file 12893_2024_2459_MOESM3_ESM.docx]
